# The Report of the Royal Commission on Vaccination

**Published:** 1896-09-12

**Authors:** 


### The Hospital

An Institutional Journal of

The Medical Sciences and Hospital Administration, with

AN EXTRA NURSING SUPPLEMENT.

Vol. XX.—No. 520.] [Sept. 12, 1896.

### THE REPORT OF THE ROYAL COMMISSION ON VACCINATION.

We would draw the attention of our readers to the analysis of the Report of the Royal Commission on Vaccination which we print on another page. The Report is not yet published, but, as we have been furnished with a copy for perusal, we have thought it well to place a sketch of its main provisions before our readers at the earliest possible opportunity.

The first point which comes out—the really important point, because it must be the basis of all action in the matter—is the complete vindication of the claims of vaccination to be prophylactic against small-pox. Not by one line of research alone, but by several routes, the same result is arrived at, and whether the matter be considered in the light of history, of experience, or of statistics, the protective influence of vaccination is made manifest.

How, then, about the evils which have been so loudly asserted to follow its performance? Here the Report is not so entirely encouraging. It is admitted that in a certain, although a very small, proportion of the cases, accidents do occur.

Cases have occurred in which erysipelas and inflammation, leading sometimes even to gangrene and death, have taken place, as, in fact, is only to be expected when the surroundings of so many of the little patients are considered. These facts have been specially borne in mind by the Commissioners in their recommendations, particularly in those regarding the sterilising of the instruments, the use of a separate tube for each child, the avoidance of vaccination at a time when the child's surroundings are of an insanitary nature, and the performance of the operation at the child's home, rather than at a station among a number of other children possibly tainted with various infections. But in regard to the main allegation about vaccination, viz., that it is a means of distributing syphilis the Report is clear that, while it is well-known that such an event is possible, it is of infinitely rare occurrence. Still, it is said that no degree of caution can give absolute security against it, and "absolute freedom from any risk of syphilis can only be had when calf lymph is used."

Turning now to the recommendations; they are fairly radical, and if adopted by the legislature will make a very great difference in the manner in which vaccination is carried out in this country. They amount, in fact, to an absolute reversal of the policy which has hitherto guided the Local Government Board in regard to the vaccination question. This policy can be summed up in two articles of faith: First, the use of arm to arm vaccination; second, the encouragement of public vaccinators and public vaccination stations. All this will be swept away if the conclusions of the Report are acted upon, for "in the forefront" of its recommendations is placed the use of calf lymph, then it is advised that the doctor should go to the patient instead of the child being brought to the station; it is rightly claimed that every duly qualified medical man who vaccinates a child successfully should be entitled to the fee; and finally it is recommended on the one hand that the vaccine vesicles should not be opened, "unless for some adequate reason," and, on the other, that the State should see that a supply of calf lymph is within the reach of every vaccinator.

This throws a great duty and responsibility on the general practitioners of the country, on whom undoubtedly the work of vaccination will fall; but if the recommendations should be accepted in the spirit in which they are made, the non-official vaccinator will not be without his reward. Not only will he be entitled to his fee, but "the amount of the fee to be received by the vaccinator would of course require to be determined with reference to the duties which it is proposed to impose upon him. We think the fee should be adequate to cover all these duties." Now as these duties include a visit to the child's home to perform the vaccination, an inspection on the third day, another after eight days, and another during the third week, as well as such attendance as the case may require if things take an abnormal course, it is obvious that, while from the public point of view the expense must be increased, from the professional point of view this greater payment, although for greater work done, will be sufficiently welcome; and to the general practitioner it will be additionally welcome from its distribution not being limited to the official class.

In regard to the recommendations made as to the compulsory clauses we need not say much. It is for human nature rather than for medical science to determine whether people are more easily led or driven; but we think with the Commissioners that while people, although, perhaps, only a small group of people, are willing to pay fines, to suffer distress of their goods, and even to go to prison rather than have their children vaccinated, it is useless to trust to still harsher methods of compulsion, and that vaccination will be best encouraged by minimising in every way the evils which at present attach to its performance, by making it as little inconvenient as possible to the parents, and, in fact, by giving vaccination to the poor in the same form as that in which it has always been accepted by the well-to-do, with the additional advantage of calf lymph being offered free to all.

We can then by no means agree with those who protest against the Report on the ground that while proving the efficacy of vaccination it fails to enforce its performance. When the Commissioners recommend the relaxation of a set of laws which notoriously are broken every day, they do not do so without recommending also what they hold to be a better way of attaining the end which they, equally with the makers of the old laws, have in view, *i.e.*, the encouragement and enforcement of vaccination.

